# Fatty Acid Composition of Muscle, Adipose Tissue and Liver from Muskoxen (*Ovibos moschatus*) Living in West Greenland

**DOI:** 10.1371/journal.pone.0145241

**Published:** 2015-12-17

**Authors:** Susana P. Alves, Katrine Raundrup, Ângelo Cabo, Rui J. B. Bessa, André M. Almeida

**Affiliations:** 1 CIISA/FMV–Centro de Investigação Interdisciplinar em Sanidade Animal, Faculdade de Medicina Veterinária, Universidade de Lisboa, Lisboa, Portugal; 2 Greenland Institute of Natural Resources, Nuuk, Greenland; 3 IBET–Instituto de Biologia Experimental e Tecnológica, Oeiras, Portugal; INIA, SPAIN

## Abstract

Information about lipid content and fatty acid (FA) composition of muskoxen (*Ovibos moschatos*) edible tissues is very limited in comparison to other meat sources. Thus, this work aims to present the first in-depth characterization of the FA profile of meat, subcutaneous adipose tissue and liver of muskoxen living in West Greenland. Furthermore, we aim to evaluate the effect of sex in the FA composition of these edible tissues. Samples from muscle (*Longissimus dorsi*), subcutaneous adipose tissue and liver were collected from female and male muskoxen, which were delivered at the butchery in Kangerlussuaq (West Greenland) during the winter hunting season. The lipid content of muscle, adipose tissue and liver averaged 284, 846 and 173 mg/g of dry tissue, respectively. This large lipid contents confirms that in late winter, when forage availability is scarce, muskoxen from West Greenland still have high fat reserves, demonstrating that they are well adapted to seasonal feed restriction. A detailed characterization of FA and dimethylacetal composition of muskoxen muscle, subcutaneous adipose tissue and liver showed that there are little differences on FA composition between sexes. Nevertheless, the 18:1*cis*-9 was the most abundant FA in muscle and adipose tissue, reaching 43% of total FA in muscle. The high content of 18:1*cis*-9 suggests that it can be selectively stored in muskoxen tissues. Regarding the nutritional composition of muskoxen edible tissues, they are not a good source of polyunsaturated FA; however, they may contribute to a higher fat intake. Information about the FA composition of muskoxen meat and liver is scarce, so this work can contribute to the characterization of the nutritional fat properties of muskoxen edible tissues and can be also useful to update food composition databases.

## Introduction

**Fig 1:**
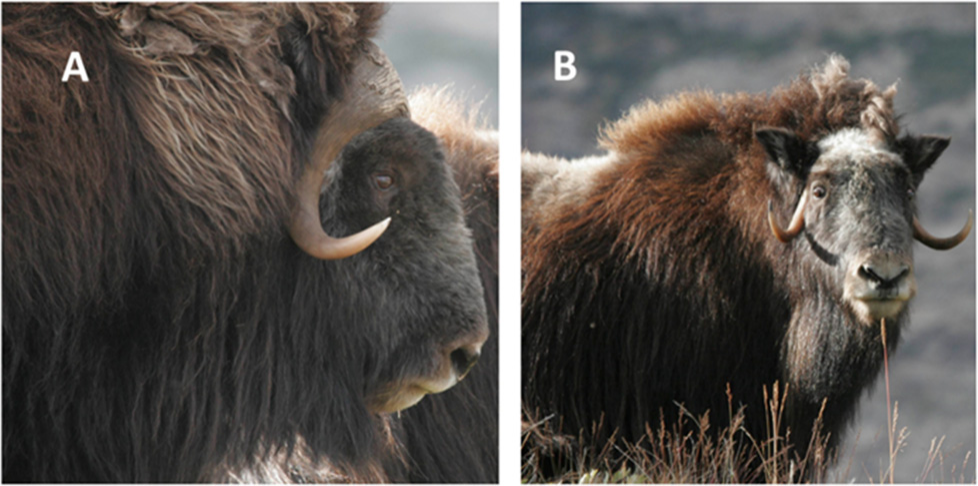
Adult male (A) and female (B) muskox (*Ovibos moschatus*) from the Kangerlussuaq-Sisimiut herd in the Angujaartorfiup Nunaa region south of Kangerlussuaq fiord (West Greenland). Photographs by K. Raundrup.

The muskox (*Ovibos moschatus*) is one of the few Pleistocene species that survived the megafaunal extinctions until present [1]. Nowadays, these large ruminants live mostly in Arctic habitats of Greenland and Northern Canada and are particularly well adapted to grazing poor-quality and frozen herbage during winter [2, 3]. Images of West Greenland muskoxen male and female are presented in Fig 1.

In West Greenland, muskoxen were introduced to the inland area Angujaartorfiup Nunaa south of the Kangerlussuaq fiord in 1962 and 1965. The introduction had two main purposes, namely to establish a new stable meat resource for hunters and further, to protect the muskoxen from extinction in North and Northeast Greenland, securing this West Greenland stock as a reserve population [4]. The founder population consisted of 27 animals that were captured in East Greenland, and following the introduction, the number of muskoxen rapidly increased in West Greenland [5]. A quota based harvesting system was established in 1988 but the muskox population still shows remarkable growth potential. In the early 2000’s the population was estimated at 7,000–10,000 animals but currently the numbers might be closer to 24,000 [6]. Muskox diet is dominated by grasses, sedges and dicots, which are low in both abundance and quality in winter [7, 8]. During summer and autumn, when high-quality forages are available, muskoxen gain large quantities of protein and fat and can retain much of this fat until late winter [9, 10], contributing to their over-winter energy needs [8, 11].

Only a few studies have reported the fat content of muskox tissues and even fewer reported the fatty acid (FA) composition of tissues. Kuhnlein et al. [12] studied the composition of the Canadian arctic traditional foods and reported that muskox raw fat and meat contained about 54% and 2% of fat on a fresh material basis, respectively. The fat content and the percentage of lipid class distribution in muskoxen from Victoria Island in northern Canada, was reported by Adamczewski et al. [11], who observed that intramuscular fat can range from 3% to 9.2% of fresh tissue. However, none of these studies reported the FA composition of adipose tissue or muscle. Indeed, as far as we know only one study has reported the FA composition of muskoxen subcutaneous adipose tissue [13]. In that study, the FA profile of muskox adipose tissue was compared with beef cattle finished on cereals in a feedlot system. The authors found that muskox adipose tissue presented higher contents of saturated FA, branch-chain FA, n-3 polyunsaturated FA and a lower n-6 to n-3 ratio compared with beef, although the FA composition of both meats were not compared or reported.

The muskox provide hides that are used in fiber production for the manufacture of traditional qiviut garments [14] and also an important source of meat. In fact, traditional Inuit food includes meat and organs, such as the liver and fat, of arctic land animals namely the muskox, moose (*Alces alces*), and caribou or reindeer (*Rangifer tarandus*) [12]. Of these foods, muskox meat has been considered of highest quality, being tender and tasty, and thus, has been requested by markets outside of the Arctic and sold mainly through websites [15, 16]. Interestingly, little is known about the lipid nutritional quality of muskox tissues such as meat, liver or adipose. Therefore, this work aims to present the first in-depth characterization of the FA profile of meat, subcutaneous adipose tissue and liver of muskoxen living in West Greenland, contributing to the characterization of the nutritional properties of this meat and valuable natural resource. Furthermore, as muskoxen are animals with a strong sexual dimorphism we will also evaluate the effect of sex on the FA composition of muscle, subcutaneous adipose tissue and liver.

## Material and Methods

### Angujaartorfiup Nunaa and Kangerlussuaq

**Fig 2:**
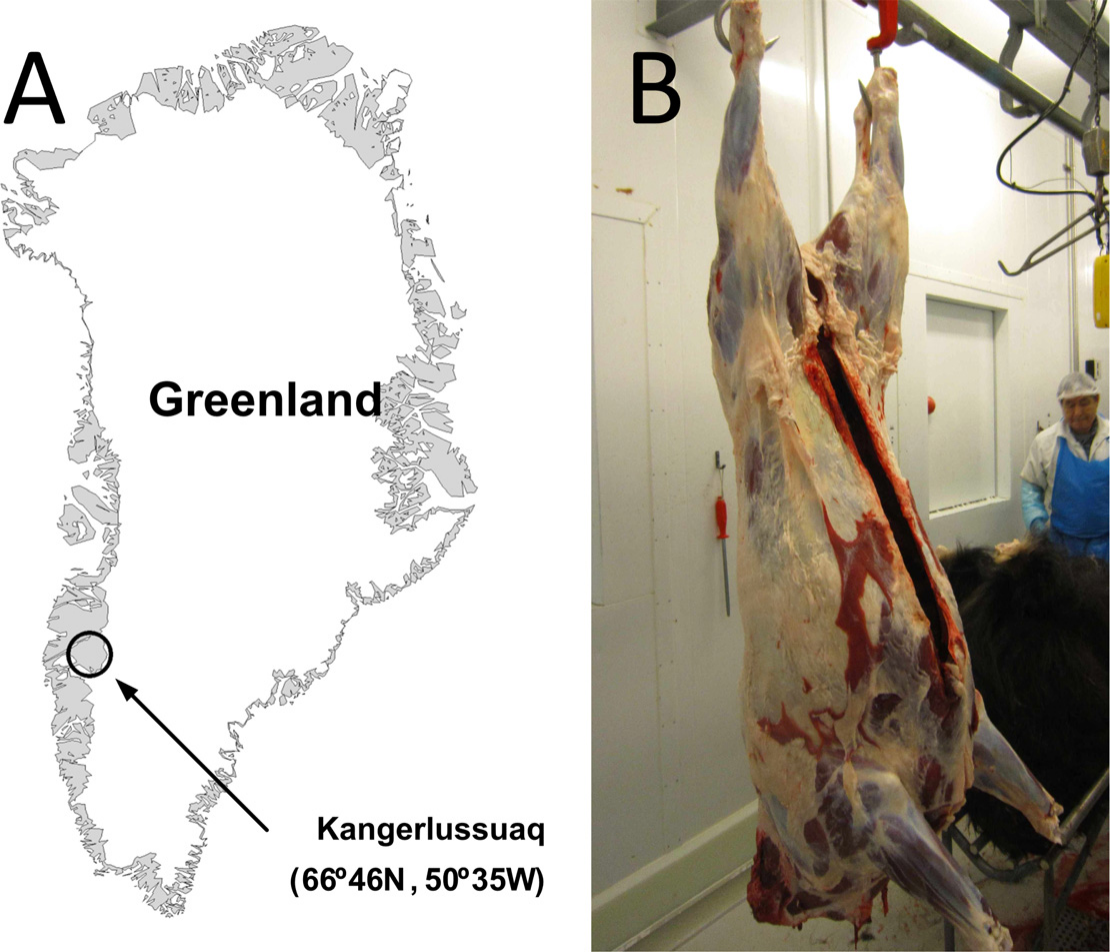
Sampling of muskox (*Ovibos moschatus*) tissues. A–Map of Greenland indicating the Kangerlussuaq region where the animals were harvested; B–Muskox carcass after skinning and evisceration. Photograph by K. Raundrup.

The inland region of Angujaartorfiup Nunaa (66°46N, 50°35W) is a 6.600 km^2^ area located south of the Kangerlussuaq fiord in West Greenland (Fig 2A). This area is characterized by a stable and dry inland climate with a yearly precipitation of approximately 150 mm. The temperatures during winter drop to daily averages of ca. -20°C with minimums of below -40°C [17, 18]. Giving the low annual precipitation, the snow cover during winter is relatively thin providing access for muskoxen and caribou to the vegetation below.

### Sample Collection

Samples from muscle (*Longissimus dorsi*), adipose tissue (rump fat) and liver were collected from female (n = 12) and male (n = 8) muskoxen in February 2014 at thebutchery in Kangerlussuaq (67°00’23.60”N, 50°41’19.71”W) (Fig 2B). Age determination was not possible for any of the animals but carcass weight (dressed weight including internal organs but without stomachs and intestine) averaged 76.0±9.51 kg for females and 89.5±19.87 kg for males. Sampling was done immediately after arrival to the butchery when the hides had been removed and before meat maturation. Approximately 5 g (wet weight) of tissue was collected per sample and stored frozen until freeze-drying. The samples were freeze-dried for a minimum of 24 hours (adipose tissue) or 72 hours (muscle and liver) until constant weight using a Christ Alpha 1–2 LD_plus_ freeze drier (Christ alpha, Osterode am Harz, Germany) at the Greenland Institute of Natural Resources, Nuuk, Greenland before further analysis.

### Ethical statement

During the quota-based hunting season in winter, muskoxen were culled and delivered to the butchery in Kangerlussuaq (Greenland) for further processing. All samples used in this study were obtained at the butchery. This study did not involve any capture of live animals or animal experimentation, thus no specific ethical approval was necessary.

### Lipid and Fatty Acid Preparation

Lipids from lyophilized muscle, adipose tissue and liver were extracted using the method of Folch et al. [19], but using dichloromethane and methanol (2:1, v/v) instead of chloroform and methanol. Total lipids were measured gravimetrically, in duplicate, by weighting the fatty residue obtained after solvent evaporation. Fatty acid methyl esters (FAME) were prepared from the lipid extracts by a basic followed by acid transesterification procedure adapted from Cruz-Hernandez et al. [20]. Briefly, 1 mL of toluene was added to the lipid extract, then 3 mL of sodium methoxide in methanol (0.5M) was added and after reaction for about 30 min at 50°C, another 2 mL of HCl in methanol (1.25M) was added to the reaction vessel, which was left to react for more 10 min at 80°C. After cooling, 2 mL of 6% aqueous potassium carbonate was added to the reaction tube and FAME were extracted with 6 mL of hexane. The solvent was removed under a flow of nitrogen at 37°C and the final residue was dissolved in 1.5 mL of hexane, and stored at -20°C until gas chromatography (GC) analysis. Additionally, a few samples (1–2 mg FAME) were hydrogenated with 1.5 mg of Adams catalyst (platinum dioxide) in 1 mL of methanol under hydrogen at 50°C for 1 hour, after cooling samples were filtrated to remove the catalyst.

### Gas Chromatography Analysis

FAME were quantified by GC with flame ionization detection (GC-FID) using a Shimadzu GC-2010 Plus chromatograph (Shimadzu, Kyoto, Japan) equipped with a 100% cyanopropyl polysiloxane capillary column (TR-CN100, 100 m, 0.25 mm i.d., 0.20 μm film thickness, Teknokroma, Barcelona, Spain). Helium was used as carrier gas at constant flow of 1 mL/min, and the injector and detector temperatures were 250 and 280°C, respectively. Column oven programmed temperature were as follows: initial oven temperature of 50°C was held for 1 min, increased to 150°C at 50°C/min and held for 20 min, then increased to 190°C at 1°C/min, and finally increased to 220°C at 2°C/min and maintained for 18 min. Identification of FAME was achieved by comparison of the FAME retention times with those of authentic standards (FAME mix 37 components from Supelco Inc., Bellefont, PA, USA). Additional identification of the FAME was achieved by electron impact mass spectrometry using a Shimadzu GC-MS QP2010 Plus (Shimadzu, Kyoto, Japan) equipped with a 100% cyanopropyl polysiloxane capillary column (SP-2560, 100 m, 0.25 mm i.d., 0.20 μm, film thickness, Supelco Inc., Bellefont, PA, USA). The GC conditions were similar to the GC-FID conditions. The mass spectrometer conditions were as follows: ion source temperature, 200°C; interface temperature, 220°C; ionization energy, 70 eV; scan, 50–500 atomic mass units.

The FAME and dimethylacetal (DMA) composition were expressed as % of total compounds (FAME+DMA), data sets are presented as supporting information (S1 Dataset). The FA sums with nutritional interest were expressed as g/100 g of dry tissue calculated considering the USDA lipid conversion factors [21]. FAME were categorized according to their chain length and structure, namely as: saturated (SFA), if they do not contain any unsaturated double bond and any methyl branched; branched chain (BCFA), if they contain a methyl group in the last carbon (iso-) or in the penultimate carbon (anteiso-); non-terminal BCFA (NT-BCFA), if they contain at least one methyl group along the carbon chain; monounsaturated (MUFA), if they contain one double bond; polyunsaturated (PUFA), if they contain more than one double bond. The FA notation contains: the number of carbon atoms in the FA chain (the number before the colon), the number of double bonds (the number after the colon), and the double bond geometry (*cis* or *trans*). The notation “n” was used for the FA recognized to belong to the n-3, n-6 and n-9 family, where the n- number indicates the position of the first double bond counted from the methyl terminal end of the carbon chain.

### Stearoyl-CoA desaturase activity indices

Stearoyl-CoA desaturase (SCD) activity indices were estimated by computing the ratio of product / (substrate + product) in both muscle and subcutaneous adipose tissue from muskoxen: SCDi-14, (14:1/(14:1+14:0)*100); SCDi-16, (16:1/(16:1+16:0)*100); SCDi-17, (17:1/(17:1+17:0)*100); SCDi-18, (18:1/(18:1+18:0)*100).

### Statistical analysis

The FA and DMA composition in muscle, adipose tissue and liver were analyzed using the PROC MIXED from SAS (SAS Institute Inc., Cary, NC, USA) with a model that included the sex (female vs. male) as the single effect. In addition, differences on the FA sums among tissues were analyzed considering the tissue as a single effect. Significance was declared at P ≤ 0.05 and data is presented as least square means and standard error of the mean (SEM).

## Results

### Fatty acid composition of muscle

**Fig 3:**
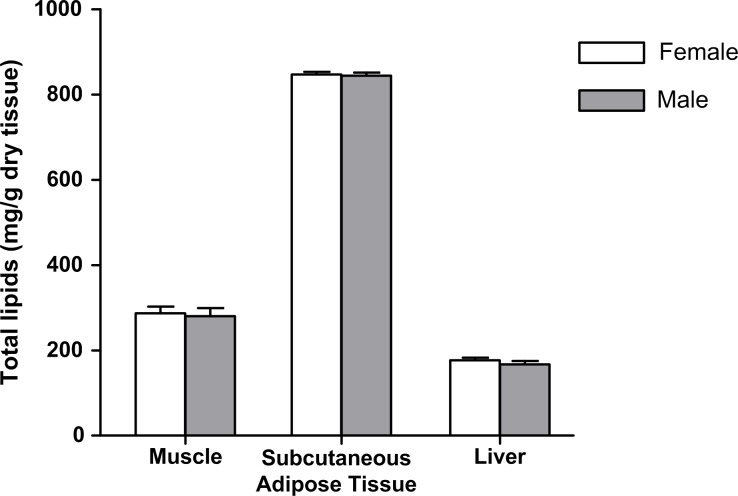
Total lipids (mg/g of dry tissue) in muscle, subcutaneous adipose tissue and liver of female and male muskoxen (*Ovibos moschatos*) in West Greenland.

Muskoxen *Longissimus dorsi* contained about 284 mg/g DM of intramuscular fat (Fig 3), with no differences between sexes (*P* = 0.799). Regarding the FA composition, more than 60 FA were identified in muscle (Table 1), with the 18:1*cis*-9 (oleic acid) being the most abundant in both sexes, comprising more than 43% of total FA in muscle. The 18:1*cis*-9 together with the 16:0 (palmitic acid) and 18:0 (stearic acid) comprise more than 87% of total FA in muscle. Although there were no significant differences between sexes on the major FA, some minor FA presented higher proportions (*P* < 0.05) in muscle of males when compared to females. These differences were observed in almost all BCFA, i.e. iso-14:0, iso-15:0, anteiso-15:0, iso-16:0, anteiso-17:0 and iso-18:0, in minor biohydrogenation intermediates (i.e. 18:2*trans-*9,*trans-*12, 18:2*cis-*9,*trans-*11 and 18:3*cis-*9,*trans-*11,*cis-*15) and also the 19:1*cis-*9 and 21:0. Several long-chain PUFA (LC-PUFA) were detected in muskox muscle, with the 20:4n-6 (arachidonic acid) and the 22:5n-3 (docosapentaenoic acid, DPA) being the most abundant in both sexes. Three DMA derived from plasmalogen lipids were also detected (i.e. 16:0, 18:0 and 18:1) in muskox muscle samples, with DMA-16:0 the most abundant DMA in both sexes (around 0.29%).

**Table 1:** Fatty acid and dimethylacetal composition (% of total compounds) in muscle of male and female muskoxen (*Ovibos moschatus*).

Muscle	Female	SEM	Male	SEM	*P* value
*Saturated and branched chain FA*					
10:0	0.05	± 0.003	0.06	± 0.004	0.541
12:0	0.02	± 0.002	0.02	± 0.002	0.575
14:0	1.44	± 0.069	1.48	± 0.085	0.771
14:0-12Me (iso)	0.02	± 0.001	0.03	± 0.002	0.001
14:0-6Me	0.005	± 0.0007	0.004	± 0.0008	0.570
14:0-8Me	0.002	± 0.0004	0.003	± 0.005	0.765
14:0-4Me	0.01	± 0.001	0.01	± 0.001	0.732
14:0-10Me	0.01	± 0.001	0.01	± 0.002	0.510
15:0	0.17	± 0.010	0.21	± 0.012	0.029
15:0-13Me (iso)	0.08	± 0.005	0.11	± 0.006	0.001
15:0-12Me (anteiso)	0.09	± 0.005	0.12	± 0.007	0.002
16:0	22.1	± 0.41	21.7	± 0.51	0.494
16:0-14Me (iso)	0.09	± 0.005	0.11	± 0.006	0.003
16:0-2Me	0.02	± 0.001	0.02	± 0.002	0.267
16:0-6Me	0.01	± 0.001	0.01	± 0.002	0.345
16:0-8Me	0.01	± 0.001	0.01	± 0.001	0.165
16:0-4Me	0.01	± 0.001	0.02	± 0.001	0.156
16:0-12Me	0.02	± 0.002	0.02	± 0.002	0.799
16:0–3,7,11,15Me	0.09	± 0.005	0.10	± 0.006	0.707
17:0	0.86	± 0.028	0.92	± 0.034	0.146
17:0-15Me (iso)	0.18	± 0.008	0.20	± 0.010	0.066
17:0-14Me (anteiso)	0.37	± 0.010	0.44	± 0.013	0.001
Cyclo-17 ^1^	0.06	± 0.004	0.07	± 0.004	0.316
18:0	22.3	± 0.47	22.2	± 0.57	0.922
18:0-16Me (iso)	0.12	± 0.005	0.14	± 0.006	0.039
20:0	0.21	± 0.013	0.24	± 0.016	0.194
21:0	0.01	± 0.002	0.02	± 0.002	0.049
22:0	0.05	± 0.006	0.06	± 0.007	0.284
24:0	0.04	± 0.004	0.04	± 0.005	0.976
26:0	0.03	± 0.003	0.02	± 0.004	0.239
*Monounsaturated FA*					
14:1 *cis-*9	0.07	± 0.005	0.08	± 0.007	0.578
16:1 *cis-*7	0.22	± 0.008	0.24	± 0.010	0.054
16:1 *cis-*9	1.66	± 0.062	1.71	± 0.076	0.596
17:1 *cis-*9	0.44	± 0.013	0.47	± 0.016	0.165
18:1 *trans-*6/-7/-8	0.03	± 0.002	0.03	± 0.003	0.554
18:1 *trans-*9	0.15	± 0.007	0.16	± 0.008	0.440
18:1 *trans-*10/-*t*11	0.48	± 0.041	0.59	± 0.050	0.132
18:1 *cis-*9 ^2^	43.3	± 0.60	43.2	± 0.74	0.890
18:1 *cis-*11 ^3^	0.92	± 0.032	0.92	± 0.039	0.972
18:1 *cis-*12	0.03	± 0.002	0.03	± 0.002	0.938
18:1 *cis-*13	0.10	± 0.007	0.10	± 0.009	0.803
18:1 *cis-*14/*trans-*16	0.03	± 0.001	0.03	± 0.001	0.861
18:1 *cis-*15	0.07	± 0.006	0.08	± 0.008	0.217
19:1 *cis-*9 ^4^	0.09	± 0.002	0.10	± 0.003	0.003
19:1 *cis-*11 ^4^	0.03	± 0.002	0.03	± 0.002	0.630
20:1	0.04	± 0.003	0.04	± 0.003	0.085
*Polyunsaturated FA*					
18:2 n-6	1.28	± 0.137	1.24	± 0.168	0.828
18:2 *trans-*11,*trans-*15	0.04	± 0.002	0.04	± 0.002	0.419
18:2 *trans-*9,*trans-*12	0.03	± 0.001	0.04	± 0.002	0.003
18:2 *cis-*9,*trans-*13/*trans-*8,*cis-*12	0.03	± 0.004	0.03	± 0.006	0.573
18:2 *trans-*8,*cis-*13/*cis-*9,*trans-*12	0.02	± 0.005	0.02	± 0.006	0.961
18:2 *trans-*9,*cis-*12	0.01	± 0.002	0.01	± 0.002	0.987
18:2 *trans-*11,*cis-*15	0.02	± 0.002	0.03	± 0.003	0.053
18:2 *cis-*9,*trans-*11 (CLA)	0.45	± 0.022	0.53	± 0.026	0.045
18:3 n-3	0.41	± 0.036	0.44	± 0.044	0.566
18:3 *cis-*9,*trans-*11,*cis-*15	0.01	± 0.003	0.03	± 0.004	0.038
20:3 n-9	0.08	± 0.008	0.07	± 0.010	0.320
20:3 n-6	0.04	± 0.004	0.04	± 0.005	0.702
20:4 n-6	0.36	± 0.044	0.34	± 0.054	0.786
20:5 n-3	0.08	± 0.011	0.08	± 0.013	0.907
22:4 n-6	0.05	± 0.003	0.04	± 0.004	0.289
22:5 n-3	0.28	± 0.021	0.28	± 0.026	0.941
22:6 n-3	0.01	± 0.002	0.01	± 0.002	0.113
*Dimethylacetals*					
16:0	0.31	± 0.033	0.27	± 0.041	0.478
18:0	0.27	± 0.038	0.26	± 0.047	0.832
18:1 *cis-*9 ^4^	0.05	± 0.010	0.07	± 0.012	0.256

Abbreviations: SEM, standard error of the mean

^1^11-cyclohexyl-11:0

^2^Contains 18:1 *cis*-10/*trans*-13/*trans*-14 as minor components

^3^Contains 18:1 *trans*-15 as minor component

^4^Tentative identification.

### Fatty acid composition of subcutaneous adipose tissue

The lipid content of the muskox subcutaneous adipose tissue is presented in Fig 3. It reached about 846 mg/g DM with no differences (*P >* 0.05) between sexes. Regarding the FA composition, the major FA in both females and males was the 18:1*cis-*9 (Table 2), however females showed a larger (*P* = 0.014) proportion compared to males, i.e. 35.8 and 32.5 g/100 g of total FA, respectively. Despite the higher proportion of 18:1*cis-*9 in females subcutaneous fat, males showed higher proportions of all remaining FA that were affected (*P <* 0.05) by sex. In particular, on all BCFA (except iso-18:0), a few SFA and MUFA (12:0, 15:0, 17:0 and 16:1*cis-*7), and a few C18 FA (18:1*trans-*10/*trans-*11, 18:1*cis-*12, 18:2*trans-*9,*trans-*12, 18:2*trans-*11,*cis-*15 and 18:2n-6). No DMA were detected in subcutaneous fat, but several NT-BCFA were detected in both females and males, comprising about 0.4% of total FA. The presence of the NT-BCFA was confirmed by mass spectrometry after and previous catalytic hydrogenation of the FAME extract.

**Table 2:** Fatty acid composition (% of total fatty acids) in subcutaneous adipose tissue of male and female muskoxen (*Ovibos moschatus*).

Adipose tissue	Female	SEM	Male	SEM	*P* value
*Saturated and branched chain FA*					
10:0	0.06	± 0.004	0.07	± 0.005	0.083
12:0	0.04	± 0.002	0.05	± 0.003	0.017
14:0	2.96	± 0.100	3.25	± 0.122	0.082
14:0-12Me (iso)	0.06	± 0.005	0.09	± 0.006	0.001
14:0-6Me	0.01	± 0.001	0.01	± 0.001	0.183
14:0-8Me	0.004	± 0.0004	0.004	± 0.0005	0.678
14:0-4Me	0.01	± 0.001	0.01	± 0.001	0.155
14:0-10Me	0.002	± 0.0004	0.002	± 0.0005	0.420
15:0	0.38	± 0.020	0.49	± 0.024	0.002
15:0-13Me (iso)	0.27	± 0.017	0.35	± 0.021	0.011
15:0-12Me (anteiso)	0.25	± 0.017	0.35	± 0.021	0.002
16:0	23.3	± 0.54	23.5	± 0.67	0.798
16:0-14Me (iso)	0.23	± 0.012	0.29	± 0.014	0.004
16:0-2Me	0.03	± 0.003	0.03	± 0.003	0.707
16:0-6Me	0.01	± 0.001	0.01	± 0.001	0.079
16:0-8Me	0.01	± 0.001	0.01	± 0.001	0.141
16:0-4Me	0.03	± 0.001	0.04	± 0.001	0.291
16:0-12Me	0.04	± 0.002	0.04	± 0.003	0.314
16:0–3,7,11,15Me	0.16	± 0.009	0.18	± 0.011	0.110
17:0	1.27	± 0.030	1.39	± 0.036	0.020
17:0-15Me (iso)	0.31	± 0.011	0.35	± 0.013	0.037
17:0-14Me (anteiso)	0.74	± 0.024	0.82	± 0.029	0.050
Cyclo-17 ^1^	0.10	± 0.006	0.09	± 0.008	0.427
18:0	24.5	± 0.52	26.1	± 0.63	0.067
18:0-16Me (iso)	0.16	± 0.005	0.18	± 0.006	0.138
20:0	0.57	± 0.032	0.64	± 0.039	0.171
21:0	0.06	± 0.005	0.07	± 0.006	0.084
22:0	0.24	± 0.026	0.30	± 0.032	0.144
23:0	0.06	± 0.005	0.08	± 0.007	0.101
24:0	0.13	± 0.010	0.13	± 0.012	0.774
25:0	0.01	± 0.001	0.01	± 0.001	0.963
26:0	0.08	± 0.006	0.07	± 0.007	0.357
*Monounsaturated FA*					
14:1 *cis-*9	0.19	± 0.011	0.17	± 0.014	0.337
16:1 *cis-*7	0.36	± 0.014	0.43	± 0.017	0.006
16:1 *cis-*9	2.53	± 0.119	2.19	± 0.145	0.090
17:1 *cis-*9	0.58	± 0.019	0.56	± 0.024	0.617
18:1 *trans-*6/-7/*-8*	0.02	± 0.002	0.02	± 0.002	0.215
18:1 *trans-*9	0.24	± 0.012	0.27	± 0.014	0.148
18:1 *trans-*10/-11	1.01	± 0.084	1.43	± 0.102	0.005
18:1 *cis-*9 ^2^	35.8	± 0.78	32.5	± 0.95	0.014
18:1 *cis-*11 ^3^	0.74	± 0.020	0.71	± 0.025	0.345
18:1 *cis-*12	0.04	± 0.003	0.05	± 0.003	0.027
18:1 *cis-*13	0.10	± 0.008	0.08	± 0.010	0.176
18:1 *cis-*14/*trans-*16	0.03	± 0.001	0.04	± 0.002	0.494
18:1 *cis-*15	0.16	± 0.009	0.17	± 0.011	0.486
19:1 *cis-*9 ^4^	0.09	± 0.015	0.08	± 0.018	0.835
19:1 *cis-*11 ^4^	0.03	± 0.001	0.03	± 0.001	0.728
20:1	0.10	± 0.007	0.11	± 0.008	0.541
24:1	0.04	± 0.003	0.04	± 0.003	0.604
*Polyunsaturated FA*					
18:2 n-6	0.41	± 0.022	0.48	± 0.026	0.039
18:2 *trans-*11,*trans-*15	0.05	± 0.002	0.05	± 0.002	0.287
18:2 *trans-*9,*trans-*12	0.08	± 0.004	0.10	± 0.005	0.029
18:2 *cis-*9,*trans-*13/*trans-*8,*cis-*12	0.05	± 0.003	0.04	± 0.004	0.205
18:2 *trans-*8,*cis-*13/*cis-*9,*trans-*12	0.03	± 0.002	0.02	± 0.003	0.691
18:2 *trans-*9,*cis-*12	0.02	± 0.002	0.02	± 0.002	0.437
18:2 *trans-*11,*cis-*15	0.07	± 0.006	0.09	± 0.007	0.026
18:2 *cis-*9,*trans-*11(CLA)	0.57	± 0.027	0.62	± 0.033	0.321
18:3 n-3	0.35	± 0.025	0.41	± 0.031	0.116
18:3 *cis-*9,*trans-*11,*cis-*15	0.04	± 0.009	0.07	± 0.011	0.061
20:3 n-6	0.01	± 0.001	0.01	± 0.001	0.150
20:4 n-6	0.02	± 0.003	0.03	± 0.003	0.368
22:4 n-6	0.02	± 0.001	0.02	± 0.001	0.183
22:5 n-3	0.17	± 0.010	0.18	± 0.012	0.350

Abbreviations: SEM, standard error of the mean

^1^11-cyclohexyl-11:0

^2^Contains 18:1 *cis*-10/*trans*-13/*trans*-14 as minor components

^3^Contains 18:1 *trans*-15 as minor component

^4^Tentative identification.

### Fatty acid composition of liver

Muskox liver samples contained about 172 mg/g DM of total lipids (Fig 3) with no difference (*P >* 0.05) between sexes. Considering the FA composition, the highest FA in females was the 18:1*cis-*9 (Table 3), whereas in males it was the 18:0. However, only 18:1*cis-*9 showed a significant effect (*P* = 0.046) between sexes. Muskox liver samples also contained relatively high proportions of 16:0, 20:4n-6, 18:2n-6 and 22:5n-3. Apart from the 18:1*cis-*9, only the 15:0 and the 22:6n-3 (docosahexaenoic acid, DHA) showed some significant effect (*P* < 0.05) between sexes, with males showing higher proportions compared to females (0.44% and 0.55% for 15:0, 0.68% and 0.86% for DHA, respectively). Three DMA were identified in liver samples, with DMA-18:0 being the major DMA showing 19% higher content compared to DMA 16:0 plus 18:1.

**Table 3:** Fatty acid and dimethylacetal composition (% of total compounds) in liver of male and female muskoxen (*Ovibos moschatus*).

Liver	Female	SEM	Male	SEM	*P* value
*Saturated and branched chain FA*					
10:0	0.04	± 0.003	0.05	± 0.003	0.743
12:0	0.02	± 0.002	0.01	± 0.002	0.611
14:0	0.52	± 0.040	0.54	± 0.049	0.761
14:0-12Me (iso)	0.03	± 0.003	0.03	± 0.003	0.158
15:0	0.44	± 0.022	0.55	± 0.027	0.008
15:0-13Me (iso)	0.17	± 0.018	0.23	± 0.023	0.066
15:0-12Me (anteiso)	0.25	± 0.031	0.33	± 0.039	0.096
16:0	15.9	± 0.53	14.7	± 0.65	0.165
16:0-14Me (iso)	0.17	± 0.014	0.22	± 0.018	0.054
16:0–3,7,11,15Me	0.26	± 0.022	0.29	± 0.027	0.333
17:0	1.72	± 0.062	1.86	± 0.076	0.169
17:0-15Me (iso)	0.28	± 0.015	0.29	± 0.019	0.455
17:0-14Me (anteiso)	0.68	± 0.034	0.77	± 0.041	0.089
Cyclo-17 ^1^	0.10	± 0.007	0.10	± 0.009	0.575
18:0	24.9	± 0.74	26.8	± 0.90	0.119
18:0-16Me (iso)	0.22	± 0.026	0.16	± 0.021	0.107
20:0	0.26	± 0.021	0.27	± 0.025	0.718
*Monounsaturated FA*					
14:1 *cis-*9	0.04	± 0.005	0.04	± 0.006	0.860
16:1	0.15	± 0.010	0.16	± 0.012	0.536
16:1 *cis-*7	0.50	± 0.020	0.48	± 0.025	0.545
16:1 *cis-*9	1.46	± 0.093	1.27	± 0.114	0.222
17:1 *cis-*9	0.60	± 0.030	0.60	± 0.036	0.871
18:1 *trans-*6/-7/-8	0.07	± 0.008	0.06	± 0.010	0.437
18:1 *trans-*9	0.15	± 0.007	0.17	± 0.009	0.061
18:1 *trans-*10/-11	0.84	± 0.051	0.95	± 0.063	0.208
18:1 *cis-*9 ^2^	25.7	± 0.86	22.8	± 1.05	0.046
18:1 *cis-*11 ^3^	1.28	± 0.067	1.22	± 0.082	0.527
18:1 *cis-*12	0.07	± 0.004	0.07	± 0.005	0.570
18:1 *cis-*13	0.15	± 0.019	0.14	± 0.023	0.799
18:1 *cis-*14/*trans-*16	0.10	± 0.010	0.13	± 0.013	0.059
18:1 *cis-*15	0.10	± 0.008	0.11	± 0.009	0.397
19:1 *cis-*9 ^4^	0.18	± 0.030	0.21	± 0.036	0.496
19:1 *cis-*11 ^4^	0.08	± 0.007	0.10	± 0.008	0.184
20:1	0.13	± 0.022	0.16	± 0.027	0.514
*Polyunsaturated FA*					
18:2 n-6	4.49	± 0.388	4.15	± 0.475	0.588
18:2 *trans-*11,*trans-*15	0.02	± 0.002	0.02	± 0.002	0.165
18:2 *trans-*9,*trans-*12	0.05	± 0.004	0.06	± 0.005	0.246
18:2 *trans-*8,*cis-*13/*cis-*9,*trans-*12	0.04	± 0.003	0.04	± 0.004	0.408
18:2 *trans-*9,*cis-*12	0.01	± 0.003	0.01	± 0.004	0.308
18:2 *trans-*11,*cis-*15	0.02	± 0.002	0.02	± 0.002	0.279
18:2 *cis-*9,*trans-*11 (CLA)	0.75	± 0.045	0.79	± 0.055	0.580
18:2 *t*rans,*trans* (CLA-*tt*)	0.07	± 0.007	0.06	± 0.009	0.445
18:3 n-6	0.07	± 0.007	0.07	± 0.009	0.922
18:3 n-3	1.28	± 0.073	1.39	± 0.089	0.364
20:2 n-6	0.11	± 0.008	0.13	± 0.010	0.092
20:3 n-9	1.01	± 0.105	1.00	± 0.129	0.943
20:3 n-6	0.78	± 0.047	0.89	± 0.057	0.178
20:4 n-6	6.59	± 0.376	7.50	± 0.461	0.143
20:5 n-3	1.95	± 0.104	2.14	± 0.127	0.248
22:4 n-6	0.27	± 0.014	0.29	± 0.018	0.332
22:5 n-3	4.14	± 0.220	4.53	± 0.270	0.277
22:6 n-3	0.68	± 0.052	0.86	± 0.063	0.044
*Dimethylacetals*					
16:0	0.08	± 0.013	0.08	± 0.016	0.712
18:0	0.13	± 0.018	0.12	± 0.023	0.843
18:1 *cis-*9	0.03	± 0.009	0.02	± 0.010	0.327

Abbreviations: SEM, standard error of the mean.

^1^11-cyclohexyl-11:0

^2^Contains 18:1 *cis*-10/*trans*-13/*trans*-14 as minor components

^3^Contains 18:1 *trans*-15 as minor component

^4^Tentative identification.

### Nutritional fatty acid sums and ratios

The FA sums with nutritional interest in muskox tissues are presented in Table 4. There were no relevant differences in the FA sums between females and males. As a result, data were compared considering only the type of tissue. Significant effects (*P* < 0.001) were obtained for all the FA sums tested. As expected, subcutaneous adipose tissue presented greater contents of SFA, branched chain FA, *cis*-MUFA and *trans*-FA compared to muscle and liver. In contrast, liver contained about 2- and 4-fold more contents of PUFA (including both n-3 and n-6 family) compared to muscle and adipose tissue, respectively. The DMA, which derived from membrane phospholipids, were only detected in muscle and liver, being its content 7-fold greater in muscle compared to liver. The n-6 to n-3 ratio was greatest in muscle followed by liver and subcutaneous adipose tissue. Regarding the FA partial sums in each tissue, muscle presented similar contents of SFA and MUFA (11.7 g/100 g of dry tissue), whereas in the adipose tissue the content of SFA was 41% greater compared to MUFA. Also in liver, the content of SFA was greatest followed by *cis*-MUFA and PUFA.

**Table 4:** Nutritional FA sums (g/100 g dry tissue) of muskox (*Ovibos moschatus*) tissues.

	Muscle		Subcutaneous tissue		Liver	
	Mean	SEM	Mean	SEM	Mean	SEM
DMA	0.14^a^	± 0.007	n.d.	-	0.03^b^	± 0.007
SFA	11.7^b^	± 0.48	43.5^a^	± 0.48	5.72^c^	± 0.482
BCFA	0.25^b^	± 0.038	1.74^a^	± 0.038	0.29^b^	± 0.038
NT-BCFA	0.06^b^	± 0.006	0.33^a^	± 0.006	n.d.	-
*cis*-MUFA	11.7^b^	± 0.506	31.3^a^	± 0.506	3.71^c^	± 0.506
*trans*-FA	0.22^b^	± 0.044	1.47^a^	± 0.044	0.20^b^	± 0.044
PUFA	0.77^c^	± 0.059	1.58^b^	± 0.059	2.96^a^	± 0.059
n-3 PUFA	0.19^c^	± 0.028	0.43^b^	± 0.028	1.09^a^	± 0.028
n-6 PUFA	0.40^b^	± 0.032	0.39^b^	± 0.032	1.62^a^	± 0.032
n-3 LC-PUFA	0.09^b^	± 0.021	0.14^b^	± 0.021	0.91^a^	± 0.021
n-6 LC-PUFA	0.10^b^	± 0.022	0.05^b^	± 0.022	1.05^a^	± 0.022
n-6/n-3	2.10^a^	± 0.044	0.92^c^	± 0.044	1.52^b^	± 0.044

Abbreviations: Means in the same row with different superscripts are significantly different (P < 0.05). DMA, dimethylacetals; SFA, linear chain saturated fatty acid; BCFA, terminal branched chain fatty acids (iso and anteiso); NT-BCFA, non-terminal branched chain fatty acids; *cis*-MUFA, includes all monounsaturated fatty acid with *cis* double bond; PUFA, polyunsaturated fatty acids; *trans*-FA, fatty acids with at least one *trans* double bond (except the 18:2*c*9,*t*11); n-6/n-3, ratio between fatty acids from n-6 and n-3 family.

### Stearoyl-CoA desaturase activity indices

**Fig 4:**
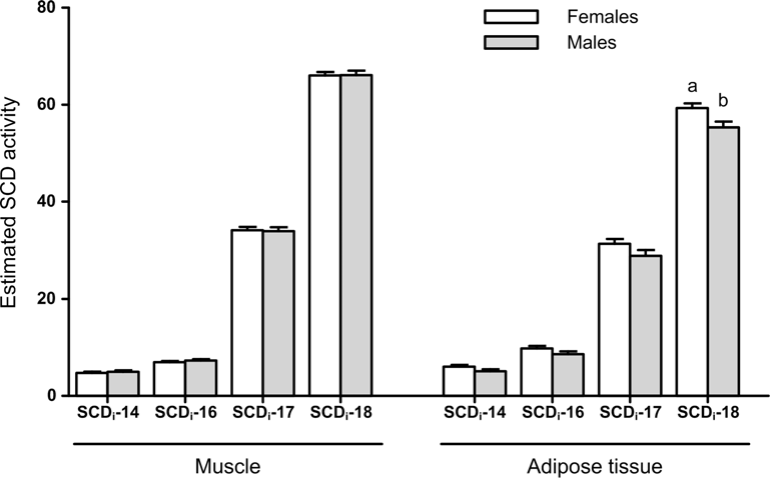
Estimated stearoyl-CoA desaturase activity indices (SCD_i_) computed from product/substrate ratios computed with the fatty acids in muscle or subcutaneous tissue. SCDi-14, (14:1/(14:1+14:0)*100); SCDi-16, (16:1/(16:1+16:0)*100); SCDi-17, (17:1/(17:1+17:0)*100); SCDi-18, (18:1/(18:1+18:0)*100).

Fig 4 presents the SCD enzyme activity indices, which is responsible for synthesizing mostly the *cis*-9 MUFA from their respective SFA. The SCD activity indices (SCD_i_) for 18:1*cis-*9 was greater in adipose tissue of muskox females compared to males (*P* = 0.02), although muscle presented a high SCD_i_-18 activity index of 66. No differences (*P* > 0.05) were observed between sexes in the other estimated SCD activity indices.

## Discussion

Muskoxen are known to accumulate body fat reserves during summer and autumn that are needed to survive the arctic winter, where the availability of high quality forage is low [8]. The samples for our study were collected during the winter hunting season, so our data suggest that in late winter, West Greenland muskoxen still have considerably high fat reserves. Indeed, our data point to an average value of 6–8% of intramuscular fat (considering a moisture content of about 75% [12]), which approaches that found in domestic ruminants [22]. However, Adamczewski et al. [11] reported that muskox intramuscular fat can reach more than 9% of fresh meat. For commercial purposes some claims have been made that muskoxen meat is paradoxically well marbled and very low in fat compared with beef, pork or poultry [15, 16]. The claim that muskoxen meat is very lean is probably based in governmental nutritional data sheets, which derived from a comprehensive characterization of the Canadian Inuit traditional food as reported by Kuhnlein et al. [12]. In that study, raw muskox meat contained only about 2% of fat per 100 g of fresh tissue. However, as data on fat content of muskox meat is scarce, the differences among these few studies might result from the time of the year where samples were collected. Indeed, it has been shown that muskox meat fat tended to increase linearly with total body fat [11], and reach the greatest value in autumn and the lowest in spring [9].

Regarding the FA composition of muscle and subcutaneous adipose tissue of muskoxen from West Greenland, it was observed that the 18:1*cis-*9 reached an extremely high content in both tissues, averaging 43% and 34% of total FA, respectively. Garton et al. [23, 24] also reported that the 18:1*cis*-9 in triglycerides of subcutaneous adipose tissues of wild animals adapted to low environmental temperatures, such as caribou or reindeer, can reach 39% and 48% of total FA, respectively. In domestic ruminants, such high concentrations in muscle and subcutaneous tissue are common in cattle finished on grain and with a greater genetic propensity to deposit fat, such as the Japanese Wagyu, resulting in meat with great fat softness and palatability [25]. Indeed, the percentage of 18:1*cis*-9 in subcutaneous adipose tissue and *Longissimus dorsi* of purebred Wagyu cattle was reported to reach 57% and 51% of total FA, respectively, [25]. In contrast, the 18:1*cis*-9 content in Longissimus muscle from genetically lean bulls raised on pasture can be as low as 20% [26, 27].

The dietary 18:1*cis-*9 is partially converted to stearic acid in the rumen due to the biohydrogenation that is carried out by the rumen microbiota. Thus, the 18:1*cis-*9, and also the 14:1*cis-*9, 16:1*cis-*9 and 17:1*cis-*9, in ruminant tissues are mainly derived from endogenous synthesis. The enzyme responsible for the introduction of the *cis*-9 double bond on the SFA is the delta-9-desaturase. This enzyme, which is encoded by the stearoyl coenzyme A desaturase (SCD) gene, may also convert the 18:1*trans-*11 to the corresponding conjugated linoleic acid (CLA) isomer, i.e. rumenic acid (18:2*cis-*9,*trans-*11 or CLA-*cis-*9,*trans-*11). The SCD product/substrate ratios computed with FA present in subcutaneous tissue or neutral muscle lipids have been used to estimate the overall SCD activity during the period of fat deposition [28]. However, wild muskoxen in West Greenland were neither produced under an intensive system nor finished on cereal grains, and because they were hunted during late winter when the quality and availability of forage is low, we can infer that the muskoxen sampled were under feed restriction. Hence, it was expected that the FA mobilization and oxidation would be increased and the lipogenesis would be reduced, presenting low SCD activity indexes. Nevertheless, the relatively high SCD indexes and the large concentration of 18:1*cis-*9 in muskoxen tissues might point to a low mobilization of the SCD products, in particular the 18:1*cis*-9. Thus, this suggests a selective salvage of the 18:1*cis*-9 during fat mobilization, which also seems more evident in females as they presented a larger proportion of 18:1*cis-*9 in subcutaneous tissue when compared to males. This might be a consequence of sexual dimorphism, where larger males should be able to withstand winter better than females. In another study, we observed that the fat tail of the Damara sheep (a breed from the fringes of the Kalahari desert) under feed restriction presented greater contents of 18:1*cis-*9 compared to controls, suggesting a similar selective mobilization [29]. Indeed, selective FA mobilization was recognized to be a general metabolic feature of adipose tissue in both rats and human [30], with the long chain SFA and MUFA being weakly mobilized compared to highly unsaturated ones [31]. Furthermore, in a study with reindeer, Soppela et al. [32] found significant reductions in the proportions of 18:2 and 18:3 in adipose tissues of starved reindeer calves (slaughtered in poor conditions in April) compared to calves slaughtered in good conditions in December. These authors also observed increased proportions of 18:1 in peristernal adipose tissue of starved reindeer calves.

There has been no information published about muskox liver lipid composition and metabolism. Although considering the liver metabolism of domestic ruminants [33], the total lipid content of muskox liver also suggests an intense fat mobilization. Indeed, in domestic ruminants, hepatic de novo FA synthesis is generally very low, so the lipid content and the FA composition in liver is greatly influenced by plasma non-esterified FA (NEFA) that are mobilized from adipose tissues [33]. Most of circulating NEFA that are captured by the liver are oxidized or esterified in triacylglycerols (TAG) to be exported in very-low-density lipoproteins (VLDL). In domestic ruminants, export of VLDL by the liver, occurs at a very slow rate relative to other species, thus under conditions of increased hepatic NEFA uptake, the TAG tend to accumulate in liver, which is known to induce the appearance of fatty liver syndrome in cows under negative energy balance [34]. The normal liver lipid content of several ruminant species (cows, buffaloes, sheep and goats) has been reported to be less than 6% of liver dry matter [35], which is far below from the 17% of total lipids in muskox dry liver. Also, the presence of relatively high proportions of 18:1*cis*-9, CLA-*cis-*9,*trans-*11, and BCFA in muskox liver, which are known to be preferentially in TAG lipids, indicates that they were possibly mobilized from adipose tissues and stored in the liver. Nevertheless, because the content and composition of TAG in the muskox livers were not determined, no specific assumption can be made.

Ruminant edible fats are known to be a good source of conjugated isomers of linoleic acid (CLA), which are a group of positional and geometric isomers of linoleic acid (18:2n-6). Some of these isomers have been shown to exert important biological effects, including anticancerinogenic and antilipogenic effects [36, 37]. In ruminant fats, the CLA-*cis-*9,*trans-*11 is the most abundant CLA isomer, which can be formed in the rumen from the biohydrogenation of dietary PUFA or can be endogenously produced in tissues from the action of the SCD on the 18:1*trans*-11. However, the content of the CLA-*cis-*9,*trans-*11 in ruminant fats depends largely on the animal’s diet. Typically, if animals are fed diets not supplemented with oils, the CLA-*cis-*9,*trans-*11 in meat can range from 0.24% to 0.85% of total FA, whereas if diets are supplemented with oils rich in PUFA, it can reach 2.4% of total FA, as reported by Aldai et al. [38]. Thus, the CLA-*cis-*9,*trans-*11 in muskox meat is comparable with that usually found in domestic ruminants not fed diets supplemented with oils. The complete CLA isomer profile of subcutaneous adipose tissue in muskoxen was reported by Dugan et al. [13], which used a combination of GC and Ag^+^-HPLC (High-Performance Liquid Chromatography). The highest CLA isomer in muskoxen adipose tissue was reported to be the CLA-*cis-*9,*trans-*11, containing about 0.34% of total FA, followed by the CLA-*trans-*11,*cis-*13, the CLA-*trans-*7,*cis-*9, the CLA-*trans-*9,*cis-*11 and other minor *trans*,*trans* CLA isomers [13]. Compared to that study, we found a fairly higher content of CLA-*cis-*9,*trans-*11 (0.6% of total FA) in subcutaneous adipose tissue of Greenland muskoxen. Nevertheless, our results can be slightly overestimated because we only used the GC analysis for CLA separation, which does not separate the CLA-*cis-*9,*trans-*11 from the CLA-*trans-*7,*cis-*9. Although the CLA-*trans-*7,*cis-*9 is the second major CLA isomer usually associated to intensively produced beef [39], it should not be present in relevant amounts in wild muskoxen adipose tissue, which are supposed to forage on grasses, sedges or dicots. This is consistent with the results of Dugan et al. [13], which reported that muskox adipose tissue contained only about 0.03% of CLA-*trans-*7,*cis-*9 on total FA.

Several types of branched-chain FA were detected in muskox tissues, which can be grouped as terminal BCFA (i.e. iso and anteiso) or non-terminal BCFA according to their structure and main metabolic origin. Iso- and anteiso-BCFA are major components of bacteria membrane lipids, and are synthesized from the branched-chain amino acids and their corresponding branched- short-chain carboxylic acids [40]. Bacteria in the rumen are known to contain several terminal BCFA, which can be further absorbed and deposited in tissues, mostly in TAG. Therefore, adipose tissues and milk lipids from ruminants are described to contain relevant amounts of iso- and anteiso-BCFA [40, 41]. In bovine adipose tissues, we found that BCFA in subcutaneous fat can range from 2.1% to 2.8% of total FA and in mesenteric fat they can reach 3.2% of total FA [42]. Similar concentrations were found in subcutaneous adipose tissue of Canadian muskoxen (2.4% of total FA) [13]. The concentration of the BCFA in subcutaneous adipose tissue in Greenlandic muskoxen was in the same range. Although there were no previous information about the concentrations of iso- and anteiso-BCFA in muskox muscle, they seem to be similar to those found in other domestic ruminants. Indeed, in several experiments conducted in our lab, we have found that iso- and anteiso-BCFA range from 0.9 to 1.6% in beef muscle [43–45] and 0.7 to 1.67% of total FA in lamb meat [29, 46, 47]. In muscle and also in adipose tissue, there were some differences in the proportion of the iso- and anteiso-BCFA between muskox sexes with females presenting lower contents compared to males. This difference might be related with the increased proportion of 18:1*cis*-9 in females, and thus because data are in weight percentages, the BCFA will be diluted by increasing the 18:1*cis*-9. In fact, when data was analyzed in mg per g of tissue, there were no differences between sexes.

In liver, the great content of BCFA, which ranged from 2.1% to 2.5% of total FA, supports the suggestion that during fat mobilization muskoxen deposited high levels of TAG in liver. Nevertheless, to the best of our knowledge, the concentration of terminal BCFA in muskox liver has not been reported so far. The importance on these BCFA in foods has been increasing in the last years because it has been reported that iso- and anteiso-BCFA might have some positive biological effects. In fact, it was verified that they can reduce the incidence of necrotising enterocolitis in rats [44], induce apoptosis in human breast cancer cells [45], and inhibit tumor growth in cultured cells and in mouse models [46]. The potential health effects of the methyl NT-BCFA, which in contrast to the iso- and anteiso-BCFA are mostly synthesized in tissues [48, 49], have not been reported. Nevertheless, their concentration in muskox adipose tissue and muscle was relatively low compared with the terminal BCFA. In contrast, high concentrations of NT-BCFA have been reported in soft subcutaneous adipose tissue of fast growing lambs fed diets containing high concentrations of easily fermentable carbohydrates [50–52] or in the fat tail Damara breed [29].

Regarding the essential FA, we found lower proportions of both 18:2n-6 and 18:3n-3 in subcutaneous adipose tissue compared to those reported the Canadian muskox [13], which might indicate a great FA mobilization of these FA in our animals, as already discussed, or to differences on the dietary FA content. There is no previous information on the contents of 18:2n-6 and 18:3n-3 in muskox muscle and liver, nevertheless the concentrations of both FA were relatively low compared with those generally found in cattle and sheep muscle [53], which again is consistent with high content of TAG in muscle and liver. Although 18:2n-6 and 18:3n-3 cannot be synthesized in tissues, they are precursors of n-6 and n-3 LC-PUFA, respectively, which are generated through elongation and desaturation by specific enzymes. The n-3 LC-PUFA, in particular EPA and DHA have been particularly recommended for consumption due to their beneficial effects to humans, which includes anti-atherogenic, anti-thrombotic and anti-inflammatory properties [54]. The DPA was the most abundant n-3 LC-PUFA in muskox samples, comprising about 76% and 61% of total n-3 LC-PUFA in meat and liver, respectively. The limited availability of high purity DPA has limiting research into its biological effects although the available data suggests that it might also has beneficial health effect to humans [55]. Dietary n-3 LC-PUFA sources for humans are mostly confined to foods of marine origin, with ruminant fats being a minor source. Even so, the liver of domestic and wild ruminants contains the highest content of LC-PUFA followed by muscle and adipose tissues [56, 57], which is consistent with our data.

The DMA composition of muskox muscle and liver has not been reported so far. Nevertheless, their composition is quite similar to that reported for muscle of domestic ruminants [29, 58]. DMA are generated from the vinyl chain linked at sn-1 position of plasmalogens, which are a particular class of glycerophospholipids present in cell membranes. Plasmalogens are widely found in anaerobic bacteria and in vertebrate animal species, where they are particularly enriched in brain, kidney, lung, and skeletal muscle, with the lowest amounts found in liver [59]. Consistent with this, reports on the DMA composition of ruminant liver are generally not reported. Moreover, we found great proportions of DMA in muskox muscle compared with liver, and because in adipose tissue the phospholipids are greatly diluted by TAG, the absence of DMA in adipose tissue is expected. In muscle, we found a lower proportion of DMA compared with other ruminants [26, 29, 60], which is consistent with the high intramuscular fat content. Indeed, as recently highlighted by Bessa et al. [28], when interpreting DMA data in muscle using its weight percentages, the DMA will be diluted by increasing neutral lipid deposition. The importance of these lipids in foods and their intake by humans should be further explored, because despite being important components of cell membranes, they offer increased antioxidant protection to PUFA and their deficiency in humans is associated to some disease states [59].

Muskox meat is not usually eaten by general consumers, but their edible tissues make part of the traditional diet in several regions across the Arctic. Meat from domestic ruminants fed on grass is usually associated with a healthier FA profile, particularly due to its n-3 LC-PUFA and CLA-*cis*-9,*trans*-11 content [61]. Nevertheless, there is no information about the nutritional quality of muskox edible fats. Regarding the n–6 to n–3 ratio, which has been used to evaluate the nutritional value of fat for human consumption, we found ratios below 2.5. Low n-6 to n-3 ratios are also commonly reported for meat of domestic ruminants fed grass diets [27, 57, 62], and also for other arctic ruminants [63], or African game meat [64]. A lower ratio has been recommended under the assumption that higher intakes of n–6 PUFA may reduce the formation of anti-inflammatory mediators from n–3 PUFA [65, 66]. However, recent conclusions of the FAO/WHO experts do not recommend a specific n-6 to n-3 PUFA ratio intake, if the intakes of n-6 and n-3 PUFA lie within their recommendations, which for a normal adult should be between 2.5–9% and 0.5–2% of energy (E) intake per day, respectively. The n-3 PUFA should also supply 0.25 to 2 g per day of the combined EPA and DHA [67].

Regarding the n-6 and n-3 PUFA concentration of muskox tissues, if we estimate a daily consumption of 100 g (considering a dry matter content of about 25% for meat and 28% for liver [12]) and a 2,000 calorie diet, meat will provide 47.5 mg (0.02% E) of n-3 PUFA and 100 mg (0.05% E) of n-6 PUFA per day, and liver will provide 305 mg (0.1% E) of n-3 PUFA and 451 mg (0.2% E) of n-6 PUFA per day. Regarding the consumption of EPA and DHA, muskox meat will provide only 5.4 mg per day, whereas liver will provide 100 mg per day, which is about 40% of the recommended intake for a normal adult (i.e. 250 mg/d [67]). Moreover, a consumption of 100 g of muskox meat and liver will provide about 16.6 mg and 155 mg of DPA per day, respectively. There is no specific dietary recommendation for DPA by the FAO/WHO experts. Nevertheless, data from the Australian population showed that DPA contributes almost 30% of total n-3 LC-PUFA to adults diet [68], with ruminant meat being the major source. In this regard, Australian and New Zealand health Authorities included the DPA in the total n-3 LC-PUFA adequate intake, which for a men should be 160 mg and for a woman should be 90 mg per day [69]. While muskox edible tissues are not good sources of PUFA, marine mammals and seafood provide good sources of n-3 PUFA, and since the latter foods are part of the traditional diet across the Arctic, the consumption of n-3 PUFA might not be an issue in Arctic regions. However, edible tissues in muskoxen can contribute to a higher fat intake. Indeed, a daily consumption of 100 g of muskox meat or liver would provide between 3.8–9.8 g (1.7–4.4% E/day) and 4.0–6.5 g (1.8–3% of E/day) of total fat, respectively, being the recommendations of FAO/WHO for the maximum total fat intake of 30–35% of energy [67]. Greenland is covered by the recommendations outlined by the Nordic Nutrition Recommendations 2004 [70], which established that fat should provide 25–35 E% for adults and children from 2 years of age.

In summary, our data showed that despite muskoxen present sex dimorphism there is no great difference on the fatty acid composition of muscle, adipose tissue or liver between females and males. Nevertheless, we confirmed that West Greenland muskoxen are able to survive the winter in good body condition and that the changes in the FA composition are probably due to selective FA mobilization, which is a characteristic of animals adapted to feed restriction. Future work should be done to the determine if during summer and autumn, where the fat deposition occurs, the FA composition of muscle, adipose tissue and liver will be distinct from the winter, or also from the late winter / spring when muskoxen are likely to be in poor body conditions. Furthermore, because information about the FA composition of muskox edible tissues and their nutritional fat quality is absent or limited, we provide data that can be useful to update the food composition databases.

## Supporting Information

S1 Dataset Lipid (mg/g DM) and fatty acid composition (% of total fatty acids) data sets of muscle, adipose tissue and liver from muskoxen females and males. (TXT)

## References

[1] Raghavan M, Espregueira Themudo G, Smith CI, Zazula G, Campos PF. Musk ox (ovibos moschatus) of the mammoth steppe: Tracing palaeodietary and palaeoenvironmental changes over the last 50,000 years using carbon and nitrogen isotopic analysis. Quaternary Sci Rev. 2014; 102: 192–201. 10.1016/j.quascirev.2014.08.001

[2] Adamczewski JZ, Chaplin RK, Schaefer JA, Flood PF. Seasonal-variation in intake and digestion of a high-roughage diet by muskoxen. Can J Anim Sci. 1994; 74: 305–13. 10.4141/cjas94-042

[3] Staaland H, Olesen CR. Muskox and caribou adaptation to grazing on the angujaartorfiup nunaa range in west greenland. Rangifer. 1992; 12: 105–13. 10.7557/2.12.2.1027

[4] Pedersen CB, Aastrup P. Muskoxen in angujaartorfiup nunaa, west greenland: Monitoring, spatial distribution, population growth, and sustainable harvest. Arctic. 2000; 53: 18–26. 10.14430/arctic830

[5] Olesen CR. Rapid population increase in an introduced muskox population, west greenland. Rangifer. 1993; 13: 27–32. 10.7557/2.13.1.1069

[6] Cuyler C, Rosing M, Molgaard H, Heinrich R, Egede J, Mathaeussen L. Incidental observations of muskox, fox, hare, ptarmigan and eagle during caribou surveys in west greenland. Technical Report No 75, 2009: Greenland Institute of Natural Resources; 2009.

[7] Nellemann C. Grazing strategies of muskoxen (ovibos moschatus) during winter in angujaartorfiup nunaa in western greenland. Can J Zool. 1997; 75: 1129–34. 10.1139/Z97-135

[8] Forchhammer MC. Sex, age, and seasonal-variation in the foraging dynamics of muskoxen, ovibos-moschatus, in greenland. Can J Zool. 1995; 73: 1344–61. 10.1139/Z95-158

[9] Adamczewski JZ, Flood PF, Gunn A. Seasonal patterns in body composition and reproduction of female muskoxen (ovibos moschatus). J Zool. 1997; 241: 245–69. 10.1111/j.1469-7998.1997.tb01956.x

[10] Thing H, Klein DR, Jingfors K, Holt S. Ecology of muskoxen in jameson land, northeast greenland. Ecography. 1987; 10: 95–103. 10.1111/j.1600-0587.1987.tb00744.x

[11] Adamczewski JZ, Flood PF, Gunn A. Body composition of muskoxen (ovibos moschatus) and its estimation from condition index and mass measurements. Can J Zool. 1995; 73: 2021–34. 10.1139/Z95-238

[12] Kuhnlein HV, Chan HM, Leggee D, Barthet V. Macronutrient, mineral and fatty acid composition of canadian arctic traditional food. Journal of Food Composition and Analysis. 2002; 15: 545–66. 10.1006/jfca.2002.1066

[13] Dugan MER, Kramer JKG, Robertson WM, Meadus WJ, Aldai N, Rolland DC. Comparing subcutaneous adipose tissue in beef and muskox with emphasis on trans 18: 1 and conjugated linoleic acids. Lipids. 2007; 42: 509–18. 10.1007/s11745-007-3051-7 17492324

[14] Rowell JE, Lupton CJ, Robertson MA, Pfeiffer FA, Nagy JA, White RG. Fiber characteristics of qiviut and guard hair from wild muskoxen (ovibos moschatus). J Anim Sci. 2001; 79: 1670–4. 11465352 10.2527/2001.7971670x

[15] Hills Foods. Wild arctic muskox [28 August 2015]. Available: http://www.hillsfoods.com/wild_game/index.php?pid=7.

[16] Nunavut Development Corporation. Nunavut muskox [28 August 2015]. Available: http://www.nunavutmuskox.ca/muskox_meat_products.html.

[17] Cappelen J, Jørgensen BV, Laursen EV, Stannius LS, Thomsen RS. The observed climate of greenland, 1958–99 –with climatological standard normal, 1961–90. Danish Meteorological Institute. 2001; Technical Report 00–18.

[18] Cappelen J. Weather observations from greenland 1958–2014. Danish Meteorological Institute. 2015; Technical Report 15–08.

[19] Folch J, Lees M, Stanley GHS. A simple method for the isolation and purification of total lipides from animal tissues. J Biol Chem. 1957; 226: 497–509. 13428781

[20] Cruz-Hernandez C, Deng ZY, Zhou JQ, Hill AR, Yurawecz MP, Delmonte P, et al Methods for analysis of conjugated linoleic acids and trans-18: 1 isomers in dairy fats by using a combination of gas chromatography, silver-ion thin-layer chromatography/gas chromatography, and silver-ion liquid chromatography. J AOAC Int. 2004; 87: 545–62. 15164853

[21] U.S. Department of Agriculture ARS. USDA national nutrient database for standard reference, release 27 Nutrient Data Laboratory Home Page Available: http://wwwarsusdagov/nutrientdata. 2014.

[22] Hocquette JF, Gondret F, Baeza E, Medale F, Jurie C, Pethick DW. Intramuscular fat content in meat-producing animals: Development, genetic and nutritional control, and identification of putative markers. Animal. 2010; 4: 303–19. 10.1017/S1751731109991091 22443885

[23] Garton GA, Duncan WRH. Fatty acid composition and intramolecular structure of triglycerides from adipose tissue of red deer and reindeer. J Sci Food Agr. 1971; 22: 29–33. 10.1002/jsfa.2740220110

[24] Garton GA, Duncan WRH, Mcewan EH. Composition of adipose tissue triglycerides of elk (cervus-canadensis), caribou (rangifer-tarandus-groenlandicus), moose (alces-alces), and white tailed deer (odocoileus-virginianus). Can J Zoolog. 1971; 49: 1159–62. 10.1139/z71-176 5113546

[25] Sturdivant CA, Lunt DK, Smith GC, Smith SB. Fatty-acid composition of subcutaneous and intramuscular adipose tissues and m-longissimus-dorsi of wagyu cattle. Meat Sci. 1992; 32: 449–58. 10.1016/0309-1740(92)90086-J 22059895

[26] Aldai N, Dugan MER, Kramer JKG, Martinez A, Lopez-Campos O, Mantecon AR, et al Length of concentrate finishing affects the fatty acid composition of grass-fed and genetically lean beef: An emphasis on trans-18:1 and conjugated linoleic acid profiles. Animal. 2011; 5: 1643–52. 10.1017/S1751731111000607 22440357

[27] Alfaia CPM, Alves SP, Martins SIV, Costa ASH, Fontes CMGA, Lemos JPC, et al Effect of the feeding system on intramuscular fatty acids and conjugated linoleic acid isomers of beef cattle, with emphasis on their nutritional value and discriminatory ability. Food Chemistry. 2009; 114: 939–46. 10.1016/j.foodchem.2008.10.041

[28] Bessa RJB, Alves SP, Santos-Silva J. Constraints and potentials for the nutritional modulation of the fatty acid composition of ruminant meat. Eur J Lipid Sci Techn. 2015: in press. 10.1002/ejlt.201400468

[29] Alves SP, Bessa RJB, Quaresma MAG, Kilminster T, Scanlon T, Oldham C, et al Does the fat tailed damara ovine breed have a distinct lipid metabolism leading to a high concentration of branched chain fatty acids in tissues? Plos One. 2013; 8 10.1371/journal.pone.0077313 PMC3800059 24204803

[30] Raclot T. Selective mobilization of fatty acids from adipose tissue triacylglycerols. Prog Lipid Res. 2003; 42: 257–88. 10.1016/s0169-7827(02)00066-8 12689620

[31] Raclot T, Groscolas R. Differential mobilization of white adipose-tissue fatty-acids according to chain-length, unsaturation, and positional isomerism. J Lipid Res. 1993; 34: 1515–26. 8228635

[32] Soppela P, Nieminen M. Effect of moderate wintertime undernutrition on fatty acid composition of adipose tissues of reindeer (rangifer tarandus tarandus l.). Comp Biochem Phys A. 2002; 132: 403–9. 10.1016/s1095-6433(02)00040-5 12020656

[33] Bell AW. Lipid metabolism in liver and selected tissues and in the whole body of ruminant animals. Prog Lipid Res. 1979; 18: 117–64. 10.1016/0163-7827(79)90013-4 396532

[34] Hocquette JF, Bauchart D. Intestinal absorption, blood transport and hepatic and muscle metabolism of fatty acids in preruminant and ruminant animals. Reprod Nutr Dev. 1999; 39: 27–48. 10.1051/rnd:19990102 10222498

[35] Tajik H, Ramin A, Nozad S, Jelodari B, Ashtab G, Eftekhari Z, et al Relationship between liver lipid and liver dry matter in slaughtered ruminants. Vet Res Forum. 2012; 3: 275–9. 25653771 PMC4313048

[36] Kelley NS, Hubbard NE, Erickson KL. Conjugated linoleic acid isomers and cancer. J Nutr. 2007; 137: 2599–607. 18029471 10.1093/jn/137.12.2599

[37] Park Y, Pariza MW. Mechanisms of body fat modulation by conjugated linoleic acid (CLA). Food Res Int. 2007; 40: 311–23. 10.1016/j.foodres.2006.11.002

[38] Aldai N, de Renobales M, Barron LJR, Kramer JKG. What are the trans fatty acids issues in foods after discontinuation of industrially produced trans fats? Ruminant products, vegetable oils, and synthetic supplements. Eur J Lipid Sci Tech. 2013; 115: 1378–401. 10.1002/ejlt.201300072

[39] Yurawecz MP, Roach JAG, Sehat N, Mossoba MM, Kramer JKG, Fritsche J, et al A new conjugated linoleic acid isomer, 7 trans, 9 cis-octadecadienoic acid, in cow milk, cheese, beef and human milk and adipose tissue. Lipids. 1998; 33: 803–9. 10.1007/s11745-998-0273-z 9727611

[40] Vlaeminck B, Fievez V, Cabrita ARJ, Fonseca AJM, Dewhurst RJ. Factors affecting odd- and branched-chain fatty acids in milk: A review. Anim Feed Sci Tech. 2006; 131: 389–417. 10.1016/j.anifeedsci.2006.06.017

[41] Ran-Ressler RR, Sim D, O'Donnell-Megaro AM, Bauman DE, Barbano DM, Brenna JT. Branched chain fatty acid content of united states retail cow's milk and implications for dietary intake. Lipids. 2011; 46: 569–76. 10.1007/s11745-011-3530-8 21293946 PMC3107348

[42] Costa ASH, Lopes PA, Estevao M, Martins SV, Alves SP, Pinto RMA, et al Contrasting cellularity and fatty acid composition in fat depots from alentejana and barrosa bovine breeds fed high and low forage diets. Int J Biol Sci. 2012; 8: 214–27. 10.7150/ijbs.8.214 22253565 PMC3258561

[43] Pestana JM, Costa ASH, Alfaia CM, Costa P, Martins SV, Alves SP, et al Lipid composition and nutritional quality of intramuscular fat in charneca-pdo beef. Eur Food Res Technol. 2012; 234: 187–96. 10.1007/s00217-011-1625-3

[44] Pestana JM, Costa ASH, Alves SP, Martins SV, Alfaia CM, Bessa RJB, et al Seasonal changes and muscle type effect on the nutritional quality of intramuscular fat in mirandesa-pdo veal. Meat Sci. 2012; 90: 819–27. 10.1016/j.meatsci.2011.11.023 22133588

[45] Pestana JM, Costa ASH, Martins SV, Alfaia CM, Alves SP, Lopes PA, et al Effect of slaughter season and muscle type on the fatty acid composition, including conjugated linoleic acid isomers, and nutritional value of intramuscular fat in organic beef. J Sci Food Agr. 2012; 92: 2428–35. 10.1002/Jsfa.5648 22473659

[46] Bessa RJB, Alves SP, Jeronimo E, Alfaia CM, Prates JAM, Santos-Silva J. Effect of lipid supplements on ruminal biohydrogenation intermediates and muscle fatty acids in lambs. Eur J Lipid Sci Tech. 2007; 109: 868–78. 10.1002/ejlt.200600311

[47] Jeronimo E, Alves SP, Martins SV, Prates JAM, Bessa RJB, Santos-Silva J. Effect of sodium bentonite and vegetable oil blend supplementation on growth, carcass quality and intramuscular fatty acid composition of lambs. Anim Feed Sci Tech. 2010; 158: 136–45. 10.1016/j.anifeedsci.2010.04.010

[48] Horning MG, Karmen A, Vagelos PR, Martin DB. Fatty acid synthesis in adipose tissue. 2. Enzymatic synthesis of branched chain and odd-numbered fatty acids. J Biol Chem. 1961; 236: 669–72. 13715907

[49] Scaife JR, Wahle KWJ, Garton GA. Utilization of methylmalonate for synthesis of branched-chain fatty-acids by preparations of chicken liver and sheep adipose-tissue. Biochem J. 1978; 176: 799–804. 747653 10.1042/bj1760799 PMC1186303

[50] Berthelot V, Normand J, Bas P, Kristensen NB. Softness and fatty acid composition of subcutaneous adipose tissue, and methylmalonic acid concentrations in the plasma of intensively reared lambs. Small Ruminant Res. 2001; 41: 29–38. 10.1016/S0921-4488(01)00190-0 11423232

[51] Garton GA, Hovell FDB, Duncan WRH. Influence of dietary volatile fatty-acids on fatty-acid composition of lamb triglycerides, with special reference to effect of propionate on presence of branched-chain components. Brit J Nutr. 1972; 28: 409–16. 10.1079/Bjn19720050 5085699

[52] Duncan WRH, Garton GA. Differences in proportions of branched-chain fatty-acids in subcutaneous triacylglycerols of barley-fed ruminants. Brit J Nutr. 1978; 40: 29–33. 10.1079/Bjn19780092 667004

[53] Wood JD, Enser M, Fisher AV, Nute GR, Sheard PR, Richardson RI, et al Fat deposition, fatty acid composition and meat quality: A review. Meat Sci. 2008; 78: 343–58. 10.1016/j.meatsci.2007.07.019 22062452

[54] Robinson JG, Stone NJ. Antiatherosclerotic and antithrombotic effects of omega-3 fatty acids. Am J Cardiol. 2006; 98: 39I–49I. 10.1016/j.amjcard.2005.12.026 16919516

[55] Dyall S. Long-chain omega-3 fatty acids and the brain: A review of the independent and shared effects of EPA, DPA and DHA. Front Aging Neurosci. 2015; 7:52 10.3389/fnagi.2015.00052 25954194 PMC4404917

[56] Kim SC, Adesogan AT, Badinga L, Staples CR. Effects of dietary n-6: n-3 fatty acid ratio on feed intake, digestibility, and fatty acid profiles of the ruminal contents, liver, and muscle of growing lambs. J Anim Sci. 2007; 85: 706–16. 10.2527/Jas.2006-289 17121972

[57] Enser M, Hallett KG, Hewett B, Fursey GAJ, Wood JD, Harrington G. The polyunsaturated fatty acid composition of beef and lamb liver. Meat Sci. 1998; 49: 321–7. 10.1016/S0309-1740(97)00143-5 22060582

[58] Rosa HJD, Rego OA, Silva CCG, Alves SP, Alfaia CMM, Prates JAM, et al Effect of corn supplementation of grass finishing of holstein bulls on fatty acid composition of meat lipids. J Anim Sci. 2014; 92: 3701–14. 10.2527/jas2013-6982 24987081

[59] Braverman NE, Moser AB. Functions of plasmalogen lipids in health and disease. Biochim Biophys Acta. 2012; 1822: 1442–52. 10.1016/j.bbadis.2012.05.008 22627108

[60] Kraft J, Kramer JKG, Schoene F, Chambers JR, Jahreis G. Extensive analysis of long-chain polyunsaturated fatty acids, CLA, trans-18: 1 isomers, and plasmalogenic lipids in different retail beef types. J Agr Food Chem. 2008; 56: 4775–82. 10.1021/Jf8001813 18491911

[61] Mapiye C, Aldai N, Turner TD, Aalhus JL, Rolland DC, Kramer JKG, et al The labile lipid fraction of meat: From perceived disease and waste to health and opportunity. Meat Sci. 2012; 92: 210–20. 10.1016/j.meatsci.2012.03.016 22546816

[62] Enser M, Hallett KG, Hewett B, Fursey GAJ, Wood JD, Harrington G. Fatty acid content and composition of uk beef and lamb muscle in relation to production system and implications for human nutrition. Meat Sci. 1998; 49: 329–41. 10.1016/S0309-1740(97)00144-7 22060583

[63] Hassan AA, Sandanger TM, Brustad M. Level of selected nutrients in meat, liver, tallow and bone marrow from semi-domesticated reindeer (*Rangifer t*. *tarandus* L.). Int J Circumpolar health. 2012; 71:17997 10.3402/ijch.v71i0.17997 22456051 PMC3417664

[64] Hoffman LC, Wiklund E. Game and venison—meat for the modern consumer. Meat Science. 2006; 74: 197–208. 10.1016/j.meatsci.2006.04.005 22062729

[65] Simopoulos AP. The importance of the omega-6/omega-3 fatty acid ratio in cardiovascular disease and other chronic diseases. Exp Biol Med. 2008; 233: 674–88. 10.3181/0711-mr-311 18408140

[66] Simopoulos AP, Leaf A, Salem N Jr. Essentiality of and recommended dietary intakes for omega-6 and omega-3 fatty acids. Ann Nutr Metab. 1999; 43: 127–30. 10436312 10.1159/000012777

[67] FAO. Fats and fatty acids in human nutrition (FAO report of an expert consultation). Rome: FAO; 2010. 21812367

[68] Howe P, Meyer B, Record S, Baghurst K. Dietary intake of long-chain ω-3 polyunsaturated fatty acids: Contribution of meat sources. Nutrition. 2006; 22: 47–53. 10.1016/j.nut.2005.05.009 16289978

[69] National health and medical research council. Nutrient reference values for australia and new zealand including recommended dietary intakes. 2006. Available: https://www.nhmrc.gov.au/_files_nhmrc/publications/attachments/n35.pdf.

[70] Becker W, Lyhne N, Pedersen AN, Aro A, Fogelholm M, Phórsdottir P, et al Nordic nutrition recommendations 2004—integrating nutrition and physical activity. Scand J Nutr. 2008; 48: 10 10.3402/fnr.v48i4.1633

